# Healthy Lifestyle Behaviour Decreasing Risks of Being Bullied, Violence and Injury

**DOI:** 10.1371/journal.pone.0001585

**Published:** 2008-02-20

**Authors:** Amelia R. Turagabeci, Keiko Nakamura, Takehito Takano

**Affiliations:** 1 International Health Section Division of Public Health, Graduate School of Tokyo Medical and Dental University, Tokyo, Japan; 2 Health Promotion Section, Division of Public Health, Graduate School of Tokyo Medical and Dental University, Tokyo, Japan; Copenhagen University Hospital, Denmark

## Abstract

**Background:**

Bullying and violence are problems of aggression in schools among adolescents. Basic daily healthy practices including nutritious diet, hygiene and physical activity are common approaches in comprehensive health promotion programs in school settings, however thier relationship to these aggressive behaviours is vague. We attempted to show the advantages of these healthy lifestyle behaviours in 9 developing countries by examining the association with being frequently bullied, violence and injury.

**Methodology/Principal Findings:**

A cross-sectional cross-national survey of 9 countries using the WHO Global School Based Student Health Survey dataset was used. Measurements included experiences of “being frequently bullied” in the preceding 30 days and violence/injury in the past 12 months. Association of risk behaviours (smoking, alcohol, sexual behaviour) and healthy lifestyle (nutrition, hygiene practices, physical activity) to being bullied, and violence/injury were assessed using multivariate logistic regression. Hygiene behaviour showed lower risks of being frequently bullied [male: RR = 0.7 (97.5CI: 0.5, 0.9); female: RR = 0.6 (0.5, 0.8)], and lower risk of experiences of violence/injury [RR = 0.7 (0.5, 0.9) for males], after controlling for risk behaviours, age, education, poverty, and country.

**Conclusion/Significance:**

Healthy lifestyle showed an association to decreased relative risk of being frequently bullied and violence/injury in developing countries. A comprehensive approach to risk and health promoting behaviours reducing bullying and violence is encouraged at school settings.

## Introduction

Bullying (occurring through interpersonal power imbalance) and violence (weighing of power resulting in injuries) are problems of aggression in schools among adolescents. These aggressive problems have recently been observed with much interest, and some schools have taken initiatives to address it to curb trends. The act of bullying includes bullies (perpetrators), victims, bullies who are victims, and observers [1], [2]; violence is usually intentional or unintentional resulting in such injuries. Victims of bullying or those being bullied are identified using questionnaires although percentages vary by studies [3]. Violence is described as a form of externalising behaviour of bullies [1], [2], [4]–[6], from complex interplay between individual, relationship, social, cultural and environmental factors described by the ecological model [7]. Due to the complexity of considering these social problems, practical definitions applicable to a school-based study have been developed.

Adolescence is a time of metamorphosis from childhood to adulthood associated with dramatic physical growth and development, as well as experimentation and acquisition of behaviours that carry high risks of morbidity and mortality. These risk behaviours such as smoking, alcohol and illicit drug use, and high-risk sexual practices [8], [9] have been shown to be associated with bullying or violence and injury [2], [10], [11], and thus influence the association between bullying and violence/injury [3]. Cessation and prevention of risk behaviours such as smoking and alcohol use, and high-risk sexual practices require a great deal of effort from individuals and advocacy groups.

In contrast, healthy lifestyle behaviours are practices carried through from childhood with which most people can identify through adolescence into adulthood. These practices include better nutritional and hygienic behaviours, and active physical activity. Healthy dietary habits are essential for oral health with stable patterns of tooth brushing established in childhood and adolescence [12]; and also known to be important for a healthy lifestyle among school children including beneficial effects on academic achievement [13], [14]. Hygiene practices are mostly associated with prevention of infectious diseases [15]. Likewise habitual physical activity in early years of life supports a healthy lifestyle [16].

The concept of a health-promoting school has been developed since the 1980s and the World Health Organization has implemented programs both in developed and developing countries [17]. Expanding children's overall capacity for health rather than increase of knowledge on a particular disease was emphasized [18]. Therefore, various behaviours of children are regarded to have a dynamic relationship. While noting the increase of antisocial behaviours of bullying and violence/injury, a wide-range of risk and health promoting behaviours relevant in developing countries have been considered, although the association among these behaviours has yet to be established. Persistence of healthy lifestyle behaviours may be a feasible and economically practical means of addressing the public health issues of bullying and violence/injury.

This study was performed to determine the associations of these risk and healthy lifestyle behaviours to experiences of being bullied and violence/injury among adolescents aged 13 to 15 years old using the WHO Global School-Based Student Health Survey (GSHS) data from 9 developing countries [China, Guyana, Jordan, Kenya, Philippine, Swaziland, Uganda, Venezuela and Zimbabwe]. It was hypothesised that while risk behaviour have a history with aggressive behaviours of bullying, and violence/injury, healthy behavioural practices may have reverse effects.

When addressing adolescent health behaviours, much focus is on addressing prevention of risk or adopted behaviours with lesser emphasis on basic daily healthy lifestyle practices. This study anticipates highlighting advantages of reinforcing healthy behavioural practices and benefits in decreasing the risks to experiences of being bullied, and experiences of violence and injuries, by respecting a holistic approach to school health based on health promoting school initiatives. The findings from this study should encourage policymakers to look beyond the traditional risk factors known to be associated with these adolescent health problems.

## Materials and Methods

### Data

Data were obtained from the 2003/2004 Global School Health Survey (GSHS), a WHO/CDC collaborative surveillance project. GSHS is a school-based survey conducted among students aged 13–15 years old to provide data on health behaviour and protective factors. The participants of the present study included 32,302 students from nine countries. For each country, the schools' response rate ranged from 90–100%, students' response rate was 76–96% and the overall response rates were 69–96%. Full details of the study, including the core questionnaire used with items selected from relevant modules, are available on the WWW at [http://www.cdc.gov/GSHS/ and http://www.who.int/chp/gshs/en/]. The protocol of sampling, survey administration, and questionnaires were pre-specified and training modules were developed. Survey coordinators participated in workshops to carry out standardized surveys.

### Measures

Age, sex, education, poverty (*measured by hunger*) [11], [19], [20], and country of the subject children were recorded according to the module. “Hunger” in this context was measured as how many days they were hungry because there was not enough food at home. The variable of country of the subject children was regarded to represent socio-cultural variance of the subjects.

Three risk behaviours and three lifestyle behaviours were measured and recorded as dichotomous variables. Risk behaviours included smoking, alcohol use and sexual behaviour. Smoking was measured using 3 items *(Q2-4 tobacco module*, Cronbach's α = .592*)* categorised as current smokers [smoked any type of cigarette once in the past 30 days, and *not* attempting to quit] and non-smokers. Alcohol use was evaluated using *(Q1,2*, α = .944*)*, as drinkers [drank alcohol from less than 1 to more than 5 drinks at least once/more days in the past 30 days] and non-drinkers. Sexual behaviour was classified as active [had sexual intercourse, multiple partners and no condom use] or inactive using items *(Q1,3,5*, α = .950*)*. Healthy lifestyle behaviours included: nutritional practices, hygiene practices, and physical activity. Nutritional practices were assessed by determining daily consumption of fruits and vegetables *(Q4,5*, α = .412*)* based on the proposed recommendations [21] [neighbouring/WHO region country used if recommendations were missing for a country]. Consumption of the recommended diet (*2 or more* servings of fruit and *3 or more* servings of vegetables per day) was classified as good nutrition and anything under the amounts stated above was classified as poor nutrition. Hygiene practices were graded as hygienic or poor hygienic behaviour, according to the practices of brushing teeth [22] and washing hands (α = .543) [23]. Physical activity was evaluated based on the first 2 questions with α = .793 with active as those who are physically active for at least 60 minutes/day in two days, and sedentary.

### Outcome Measures

According to the report on the days being bullied in the preceding 30 days and the report of ways of being bullied, we assessed subjects' status of being bullied. Satisfactions to the two independent variables were examined. The variable “being frequently bullied” was defined as those who were bullied 3 days or more in the preceding 30 days in any of the following ways: *“being hit, kicked, shoved around, or locked indoors”; “being made fun of- ‘because of my race or colour’, ‘because of my religion’, ‘ because of how my body or face looks’, or ‘ with sexual jokes, comments or gestures’ ”; “being left out of activities on purpose or completely ignored”; and “ bullied in some other way”.* The variable “ever being bullied” was defined as those who reported they were bullied at least once in the preceding 30 days, by any form of bullying. These two variables were developed according to the definition used in earlier studies [1], [2], [4], [23]. By definition, “being frequently bullied” includes “ever being bullied”. Those who were not “ever being bullied” were regarded as “being not bullied” in this study.

Violence and injury were evaluated as follows. Violence: *During the past 12 months, how many times were you in a physical fight?* This was preceded by the definition: *A physical fight occurs when two or more students of about the same strength or power choose to fight each other*. Injury was defined as: *An injury is serious when it makes you miss at least one full day of usual activities (such as school, sports or job) or requires treatment by a doctor or nurse*. This was evaluated from the question: *During the past 12 months, what were you doing when the most serious injury happened to you?*

### Analyses

The prevalence rates for being frequently bullied, ever being bullied, violence/injury, and risk and healthy lifestyle behaviours were calculated by gender. Relative risks (RRs) of being frequently bullied for violence/injury risk behaviours, and healthy lifestyle behaviours, and RRs of violence/injury for risk behaviours, and healthy lifestyle behaviours were calculated.

Multivariate logistic regression analyses were performed to calculate adjusted RRs of being frequently bullied and violence/injury by risk and healthy lifestyle behaviours. The 97.5% confidence intervals were calculated to adjust for multiple outcome assessment. Sets of independent variables used in the multivariate models were age [model I], age, education, and poverty [model II], age, education, poverty, and country [model III], and age, education, poverty, country, and alcohol and smoking behaviours [model IV]. In this analysis RRs ranges 0–1 and 1< were regarded as protective and risk effects.

Multivariate logistic regression analyses were also performed to calculate adjusted RRs of ever being bullied by risk and healthy lifestyle behaviours. The same set of additional independent variables was used for model I, II, III, and IV.

Statistical analyses were performed using SPSS version 14 (SPSS Inc., Chicago, IL, USA).

## Results

This study included a sample of 18,420 adolescent girls and 13,882 boys aged 13–15 years old from 9 countries. Table 1 shows the prevalence rates for being bullied and violence/injury as well as risk and healthy lifestyle behaviours. The prevalence rates among males for being frequently bullied 35% and violence/injury 59%, ever being bullied were 42%, and those among females were 34%, 39%, and 43%, respectively. Risk behaviours were high for boys, while girls reported high levels of nutritional and hygienic practices (67% and 69% respectively). The logistic regression analyses revealed that collectively for both sexes, the RRs of being frequently bullied was higher amongst those who were also victims of violence and injuries. Similarly, being involved in risky behaviour (i.e. smoking, alcohol and sexual activity) inferred higher RRs to being frequently bullied and being victims of violence and injuries. Contrary to that, healthy lifestyle behaviour showed lower inference with being frequently bullied and violence/injury, except for active physical activity, to being frequently bullied.

**Table 1:** Lifestyle and behaviour for schoolchildren aged 13–15 years old.

		Male	Female	Being frequently bullied				Violence & Injury			
		N (%)	N (%)	Yes (%)	No (%)	RR	*97.5%CI*	Yes (%)	No (%)	RR	*97.5%CI*
***Ever Bullied***											
**All**		**11834**	**16111**								
Not bullied		6860 (58.0)	9900 (61.4)								
Ever bullied		4974 (42.0)	6211 (38.6)								
^#^Frequently bullied		4171 (35.2)	5556 (34.5)								
***Health compromising behaviour***											
**Violence &**	**total**	**9789**	**13710**								
**Injury**	Yes	5782 (59.1)	5888 (42.9)	46.3	53.7	**2.9**	**(2.7, 3.1)**				
	No	4007 (40.9)	7822 (57.1)	23.2	76.8						
***Risk behaviours***											
**^§^Smoking**	Smoker	1319 (17.5)	711 (7.1)	48.2	51.8	**2.0**	**(1.8, 2.3)**	74.4	25.7	**3.41**	**(2.9, 4.0)**
	Non	6222 (82.5)	9244 (92.9)	31.5	68.5			45.9	54.1		
**^¶^Alcohol**	Drinker	2474 (22.6)	2435 (15.9)	45.1	54.9	**1.9**	**(1.7, 2.0)**	60.1	39.9	**1.88**	**(1.7, 2.0)**
	Non	8492 (77.4)	12842 (84.1)	30.5	69.5			44.5	55.5		
**^»^Sexual Activity**	Active	1064 (25.3)	487 (6.8)	44.5	55.5	**1.5**	**(1.3, 1.7)**	65.7	34.3	**2.16**	**(1.9, 2.5)**
	Inactive	3135 (74.7)	6723 (93.2)	34.5	65.5			47.0	53.0		
***Healthy lifestyle behaviours***											
**^¶^Nutrition**	Good	7527 (63.1)	10476 (66.9)	31.5	68.5	**0.9**	**(0.8, 1.0)**	46.3	53.7	0.98	(0.9, 1.0)
	Poor	4407 (36.9)	5191 (33.1)	33.9	66.1			46.8	53.2		
**^¶^Hygiene**	Hygienic	7700 (64.4)	11181 (69.3)	32.9	67.1	**0.6**	**(0.5, 0.7)**	45.3	54.7	**0.80**	**(0.7, 0.9)**
	Poor	4253 (35.6)	4956 (30.7)	44.9	55.1			51.6	48.4		
**^¶^Physical Activity**	Active	7056 (67.2)	8080 (61.1)	35.2	64.8	**1.1**	**(1.0, 1.1)**	48.0	52.0	**0.93**	**(0.9, 1.0)**
	Sedentary	3450 (32.8)	5147 (38.9)	33.7	66.3			49.9	50.1		

¶ Lifestyle not reported from 1 country, § Lifestyle not reported from 2 countries, »Sexual activity not reported from 4 countries.

RR - Prevalence risk ratio estimate with confidence interval

#Frequently bullied = ever bullied ≥3 days past 30 days; Bold = significant association.

Table 2 shows the results of RRs of risk and healthy lifestyle behaviours to being frequently bullied. Risk behaviours including smoking, alcohol and active sexual behaviour showed increased RRs of being frequently bullied in males and females. The RRs for hygienic behaviour to being frequently bullied remained stable from model I to IV for both males and females. The results obtained by model IV indicated independent significant associations between hygienic behaviour and being frequently bullied, after excluding the influence of age, education, poverty, country, and smoking and alcohol. The analyses using “ever being bullied” as dependent variables showed similar results as shown in the Table S1.

**Table 2:** Results of multivariate logistic regression analyses for the association of risk and healthy lifestyle behaviours to being frequently bullied among adolescents aged 13–15 years old in 9 countries.

		Being frequently bullied							
		Model I		Model II		Model III		Model IV	
		RR	97.5%CI	RR	97.5%CI	RR	97.5%CI	RR	97.5%CI
^§^Smoking	Male	**1.8**	**(1.5, 2.2)**	**1.7**	**(1.5, 2.1)**	**1.7**	**(1.5, 2.1)**		
	Female	**2.3**	**(1.8, 3.0)**	**2.2**	**(1.9, 2.5)**	**2.2**	**(1.9, 2.5)**		
^¶^Alcohol drinking	Male	**1.8**	**(1.6, 2.1)**	**1.8**	**(1.6, 2.1)**	**1.8**	**(1.6, 2.1)**		
	Female	**1.9**	**(1.7, 2.1)**	**1.9**	**(1.7, 2.1)**	**1.9**	**(1.7, 2.1)**		
^»^Active sexual behaviour	Male	**1.5**	**(1.2, 1.9)**	**1.5**	**(1.2, 1.9)**	**1.5**	**(1.3, 1.9)**		
	Female	**1.8**	**(1.4, 2.3)**	**1.8**	**(1.4, 2.3)**	**1.8**	**(1.3, 2.3)**		
^¶^Good nutrition behaviour	Male	**0.8**	**(0.7, 0.9)**	**0.9**	**(0.8, 0.9)**	**0.9**	**(0.8, 1.0)**	0.9	(0.7, 1.0)
	Female	0.9	(0.8, 1.1)	0.9	(0.8, 1.1)	0.9	(0.8, 1.1)	0.9	(0.8, 1.1)
^¶^Hygienic behaviour	Male	**0.6**	**(0.5, 0.8)**	**0.7**	**(0.5, 0.9)**	**0.7**	**(0.5, 0.9)**	**0.7**	**(0.5, 0.9)**
	Female	**0.6**	**(0.4, 0.7)**	**0.6**	**(0.4, 0.8)**	**0.6**	**(0.4, 0.8)**	**0.6**	**(0.5, 0.8)**
^¶^Physically active	Male	0.9	(0.8, 1.1)	1.0	(0.8, 1.2)	1.2	(1.0, 1.4)	**1.2**	**(1.0, 1.5)**
	Female	**0.9**	**(0.8, 1.0)**	0.9	(0.8, 1.1)	1.0	(0.9, 1.2)	1.0	(0.9, 1.2)

¶ Not reported from 1 country. § Not reported from 2 countries. » Not reported from 4 countries Model I = adjusted for age only; Model II = Model I+education+poverty; Model III = Model II+country; Model IV = Model III+smoking+alcohol; RR = Relative risk ratio; **Bold** = significant association

***Reference*** category were: *“other”* for smoking & alcohol; *“Inactive”* for sexual behaviour; *“Poor”* for hygiene & nutrition behaviour; *“sedentary”* for physical activity.

Table 3 shows the results of multivariate logistic regression analysis of risk and healthy lifestyles with violence and injury. The inferred RRs for risk behaviours to violence/injury were higher compared to associations of being frequently bullied. Smoking showed the highest relative risk; the inferred RR was highest at 2.9 for males [97.5%CI = 2.3–3.6] and was at 3.1 for females [97.5%CI = 2.3–4.1]. Compared to healthy lifestyle behaviour, among males, hygienic behaviour had low inferred associated RRs to violence/injury; a similar trend of association was notable among those with better nutritional practices. Active physical activity was associated with decreased relative risks of violence and injury among males. This table shows that healthy behaviour decreases risk of experience of violence and injuries among males in these developing countries.

**Table 3:** Results of multivariate logistic regression analyses for the association of risk and healthy lifestyle behaviours to violence/injury among adolescents aged 13–15 years old in 9 countries.

		Violence/injury							
		Model I		Model II		Model III		Model IV	
		RR	97.5%CI	RR	97.5%CI	RR	97.5%CI	RR	97.5%CI
^§^Smoking	Male	**2.8**	**(2.3, 3.5)**	**2.8**	**(2.2, 3.5)**	**2.9**	**(2.3, 3.6)**		
	Female	**3.2**	**(2.5, 4.1)**	**3.2**	**(2.4, 4.3)**	**3.1**	**(2.3, 4.1)**		
^¶^Alcohol drinking	Male	**2.2**	**(2.0, 2.6)**	**2.3**	**(2.0, 2.7)**	**2.3**	**(2.0, 2.6)**		
	Female	**1.5**	**(1.3, 1.7)**	**1.6**	**(1.4, 1.8)**	**1.5**	**(1.3, 1.7)**		
^»^Active sexual behaviour	Male	**1.7**	**(1.4, 2.1)**	**1.8**	**(1.4, 2.2)**	**1.8**	**(1.4, 2.2)**		
	Female	**1.6**	**(1.2, 2.0)**	**1.7**	**(1.3, 2.2)**	**1.7**	**(1.3, 2.2)**		
^¶^Good nutrition behaviour	Male	0.9	(0.8, 1.1)	1.0	(0.9, 1.1)	1.0	(0.9, 1.1)	0.9	(0.8, 1.1)
	Female	**1.2**	**(1.1, 1.2)**	**1.1**	**(1.0, 1.2)**	**1.2**	**(1.1, 1.3)**	1.1	(0.9, 1.3)
^¶^Hygienic behaviour	Male	**0.8**	**(0.7, 0.9)**	**0.8**	**(0.7, 1.0)**	**0.8**	**(0.7, 1.0)**	0.9	(0.6, 1.1)
	Female	0.9	(0.8, 1.1)	1.0	(0.9, 1.2)	1.1	(0.9, 1.3)	1.2	(0.9, 1.7)
^¶^Physically active	Male	**0.7**	**(0.6, 0.9)**	**0.8**	**(0.7, 1.0)**	1.1	(0.9, 1.4)	1.1	(0.9, 1.4)
	Female	1.0	(0.8, 1.1)	1.0	(0.8, 1.2)	1.0	(0.8, 1.2)	1.0	(0.8, 1.2)

¶ Not reported from 1 country. § Not reported from 2 countries. » Not reported from 4 countries.

Model I = adjusted for age only; Model II = Model I+education+poverty; Model III = Model II+country; Model IV = Model III+smoking+alcohol; RR = Relative risk ratio; **Bold** = significant association

***Reference*** category were: *“other”* for smoking & alcohol; *“Inactive”* for sexual behaviour; *“Poor”* for hygiene & nutrition behaviour; *“sedentary”* for physical activity.

## Discussion

Analysis of a cross-national survey of 9 countries using the WHO Global School Based Student Health Survey dataset indicated that “being frequently bullied” (being bullied 3 days or more in the preceding 30 days) (35% males/34% females), “ever being bullied” (being bullied at least once in the preceding 30 days) (42% males/39% females) and violence/injury (59% males/43% females) are prevalent among adolescents in these developing countries. The low risk ratios between hygienic behaviours and “being frequently bullied” among adolescents in developing countries were detected, even after controlling for socioeconomic, socio-cultural and risk behavioural factors. Similar analysis was conducted for “ever being bullied” which showed similar lower relative risks for hygienic behaviour. Risk and healthy lifestyle behaviours increased and reduced the likelihoods of being bullied and violence/injury, respectively.

The inferred associations between smoking, alcohol and active sexual behaviour to increased risks of being bullied, and violence/ injury are consistent with those reported in other studies [2], [6], [7], [9], [24] and similar for frequently bullied, reiterating the importance of addressing these issues among adolescents as these are global public health problems. Similar to the status of smoking as a gateway for illicit drug use with increased likelihood of leading to alcohol consumption in a cycle of “problem behaviour” [25], risk behaviours are regarded as having some entry relationships with being frequently bullied and violence/injury. Imitating adult-like behaviours are ways in which adolescence get to experience risk-taking lifestyle. Prevention of smoking or alcohol use during adolescence is encouraged not only to mitigate the direct adverse health influences of these behaviours but also because of their indirect relationships with social problems, including bullying and violence/injury.

The benefits of good nutrition [12]–[14] cannot be denied and trends in this study implicating decreasing relative risk ratio to being frequently bullied and experience of violence/injuries in both sexes. Good nutrition is vital for growth spurts during adolescence and while studies have shown diets low in fruits and vegetables to be associated with low academic performances, this analysis shows the assumption to low risk of being victimised. As important as it is for development, the inferences made in the current study will encourage research on other potential benefits.

Hygiene behaviour literally means personal cleanliness. Thus, adolescents labelled “dirty” by their peers are prone to being victimised or bullied, and the stigma could therefore lead to depressive moods and/or violent behaviour. From a psychological viewpoint, bullying is a type of aggression associated with behaviour intended to harm or disturb others that occurs repeatedly over time with an imbalance of power, and may be inflicted either directly (physical: overt threats, pushing) or indirectly (psychological: rumours, shunning) [2]. Thus, adolescents with poor hygiene practices are more likely to be stigmatised and this may lead to depression, which may further escalate to suicide as studies have shown an association between bullying and suicide ideation [23]. Although increased awareness of hygiene behaviour in developing countries is encouraged for prevention and control of communicable diseases, this finding infers that it may be an economically viable and effective anti-bullying activity.

Physical activity is widely recommended for intervention against obesity and for improved general health. The trend for low relative risk associated with active physical activity to being frequently bullied and in the first two models for association to violence/injury for both sexes infers a protective mechanism. Although regular exercise has been suggested to be a health-enhancing behavioural practice [26], it can be perceived that physically active students are fit and may therefore be able to protect themselves. Hart *et al.* reported that exercise has psychological benefits by improving social skills and providing a “break” from the mental exertion of academic work [13]. This inferred association could contribute to the prevention of new cases among females and may reduce the problems of existing victims, particularly in developing countries.

This is the first study with a large sample size and data drawn from a cross-national dataset indicating associations of healthy lifestyle behaviours with victims of bullying and violence/injury. When comparing the observation made by classifying victims of bullying in the strict definition by frequency of being bullied and those bullied in general, the associated relative risks shows similar observations. Risk behaviour increased the relative risk while hygienic behaviours showed lower risk, even with nutrition practice showing similar trends of lower risk of association. The observation of the problems of ever being bullied, being frequently bullied, violence and injury common across many developed countries are now observed in developing countries. The observed similarities in these developing countries suggest that consideration be given to the public health importance of being bullied, violence and injury. While healthy lifestyle behaviours are positives in reducing and preventing chronic and infectious diseases in developing countries, they are found to be supportive to victims of bullying and violence/injuries. There are several caveats that must be taken into consideration when interpreting the results of this study. The cross-sectional nature of this study precludes causal inferences. The study sample was confined to a narrow age range of 13–15 years old, and therefore the findings cannot be generalised to all adolescents.

This study shows inference of association to decreased risk of being bullied and experience of violence/injury for healthy lifestyle behaviour, the effect remaining even after controlling for risk behaviour, exhibiting a buffering effect. The low relative risk with nutrition and hygiene behaviours demonstrates the health-enhancing capacity of such practices in contrast to risk behaviours as a vulnerability gateway even to compromising behaviours. The importance of adopting a healthy lifestyle, such as good nutritional and hygienic practices, and physical activity is usually established from childhood and should be encouraged daily. It is useful to advocate such behaviours through healthy school settings as these settings are guided by the holistic approach to health and principles of equity and empowerment. Consideration of safe sex is becoming a critical issue in recent school-based health promotion for youth. The results of this study give insight into how a wide range of factors associated with being bullied, violence and injuries and help countries establish programs, and advocate for resources for school and youth health programs. Although this is not an intervention study, the results showed an association between predictive factors and outcome variables. Prospective studies are required to follow up the basic health behaviours and their influence on social and mental health problems.

## Supporting Information

Table S1 Results of multivariate logistic regression analyses for the association of risk and healthy lifestyle behaviours to ever being bullied among adolescents aged 13–15 years old in 9 countries.
(0.06 MB DOC)
