# Supplementary material for: Healthy Lifestyle Behaviour Decreasing Risks of Being Bullied, Violence and Injury
Source: PLoS One. 2008 Feb 20;3(2):e1585. doi: 10.1371/journal.pone.0001585 (PMC2249928; doi:10.1371/journal.pone.0001585)
Supplement: Table S1 — Results of multivariate logistic regression analyses for the association of risk and healthy lifestyle behaviours to ever being bullied among adolescents aged 13–15 years old in 9 countries. (0.06 MB DOC) [file pone.0001585.s001.doc]

| ***Table S1.*** Results of multivariate logistic regression analyses for the association of risk and healthy lifestyle behaviours to ever being bullied among adolescents aged 13-15 years old in 9 countries. | | | | | | | | | |
| --- | --- | --- | --- | --- | --- | --- | --- | --- | --- |
|  |  | Ever being bullied | | | | | | | |
|  |  | Model I | | Model II | | Model III | | Model IV | |
|  |  | RR | 95%CI | RR | 95%CI | RR | 95%CI | RR | 95%CI |
| ^§^Smoking | Male | **2.0** | **(1.7, 2.3)** | **1.9** | **(1.6, 2.2)** | **1.9** | **(1.6, 2.2)** |  |  |
|  | Female | **2.4** | **(1.9, 3.0)** | **2.3** | **(1.8, 2.8)** | **2.2** | **(1.8, 2.8)** |  |  |
| ^¶^Alcohol drinking | Male | **2.0** | **(1.8, 2.3)** | **2.0** | **(1.8, 2.3)** | **2.0** | **(1.8, 2.2)** |  |  |
|  | Female | **1.8** | **(1.7, 2.0)** | **1.9** | **(1.7, 2.1)** | **1.8** | **(1.7, 2.0)** |  |  |
| ^»^Active sexual behaviour | Male | **1.6** | **(1.3, 1.9)** | **1.6** | **(1.3, 1.9)** | **1.6** | **(1.3, 1.9)** |  |  |
|  | Female | **1.7** | **(1.4, 2.2)** | **1.7** | **(1.4, 2.2)** | **1.7** | **(1.4, 2.2)** |  |  |
| ^¶^Good nutrition behaviour | Male | **0.9** | **(0.8, 1.0)** | 0.9 | (0.8, 1.0) | 0.9 | (0.8, 1.0) | 0.9 | (0.8, 1.1) |
|  | Female | 0.9 | (0.8, 1.0) | 1.0 | (0.8, 1.1) | 1.0 | (0.9, 1.1) | 1.0 | (0.9, 1.1) |
| ^¶^Hygienic behaviour | Male | **0.6** | **(0.5, 0.8)** | **0.7** | **(0.5, 0.8)** | **0.6** | **(0.5, 0.8)** | **0.7** | **(0.5, 0.9)** |
|  | Female | **0.6** | **(0.5, 0.8)** | **0.6** | **(0.5, 0.8)** | **0.6** | **(0.5, 0.8)** | **0.6** | **(0.5, 0.8)** |
| ^¶^Physically active | Male | 0.9 | (0.8, 1.1) | 1.0 | (0.9, 1.2) | **1.2** | **(1.0, 1.4)** | **1.2** | **(1.0, 1.4)** |
|  | Female | **0.9** | **(0.8, 1.0)** | 0.9 | (0.8, 1.0) | 1.0 | (0.9, 1.2) | 1.0 | (0.9, 1.2) |
| ^¶^ Not reported from 1 country. § Not reported from 2 countries. » Not reported from 4 countries. | | | | | | | | | |
| Model I = adjusted for age only; Model II = Model I + education + poverty; Model III = Model II + country; Model IV = Model III + smoking + alcohol; RR = Relative risk ratio; **Bold** = significant association | | | | | | | | | |
| ***Reference*** categories were: *"other"* for smoking & alcohol; *"Inactive"* for sexual behaviour; *"Poor"* for hygiene & nutrition behaviour; *"sedentary"* for physical activity. | | | | | | | | | |
